# Mediastinal epithelioid hemangioendothelioma with abundant spindle cells and osteoclast-like giant cells mimicking malignant fibrous histiocytoma

**DOI:** 10.1186/1746-1596-8-103

**Published:** 2013-06-21

**Authors:** Xiao-Man Li, Xu-Yong Lin, Hong-Tao Xu, Juan-Han Yu, Liang Wang, Chui-Feng Fan, Yang Liu, En-Hua Wang

**Affiliations:** 1 Key Laboratory of Medical Cell Biology, Ministry of Education, China Medical University, Shenyang 110001, China; 2 Department of Pathology, the First Affiliated Hospital and College of Basic Medical Science, China Medical University, Shenyang 110001, China; 3 Institute of pathology and pathophysiology, China Medical University, Shenyang 110001, China

**Keywords:** Epithelioid hemangioendothelioma, Mediastinum, Osteoclast-like giant cells, Malignant fibrous histiocytoma

## Abstract

**Abstract:**

Epithelioid hemangioendothelioma is a relatively uncommon lesion usually presenting in soft tissues. The occurrence in the mediastinum is exceptional rare. Histologically, this tumor is characterized by epithelioid cells with intracytoplasmic vacuoles in a hyalinized or mucinous stroma. Occasionally, spindle cells or osteoclast-like giant cells can be observed. Herein, we present a case of epithelioid hemangioendothelioma in a 38 year-old Chinese male. The tumor was predominantly composed of abundant spindle cells with marked atypia and scattered osteoclast-like giant cells reminiscent of malignant fibrous histiocytoma. The unusual histological appearance can pose a great diagnostic challenge. It may be easily misdiagnosed, especially if the specimen is limited or from fine-needle aspiration.

**Virtual slides:**

http://www.diagnosticpathology.diagnomx.eu/vs/5804918529726307

## Background

Epithelioid hemangioendothelioma (EHE) is a rare vascular endothelial tumor first described by Weiss and Enzinger in 1982 [1]. It is usually considered an intermediate vascular neoplasm between a benign hemangioma and a highly aggressive angiosarcoma [2].

EHE can occur at any age, but usually in the adulthood. It can occur in soft tissues and various organs including lung, bone, liver and skin [3-5]. However, the mediastinal location is very exceptional, and only few cases were reported [6-11]. EHE is histologically characterized by cords and nests epithelioid cells with intracytoplasmic vacuoles in a myxohyaline stroma. Very rarely, the tumor may be present with spindle cells and multinucleated osteoclast-like giant cells [10,11]. Herein, we present a case of EHE in a 38-year-old Chinese male. Histologically, the tumor was predominately composed of sheets of atypical spindle cells and scattered cells reminiscent of malignant fibrous histiocytoma. The unusual histological appearance may pose a great diagnostic challenge. It may be easily misdiagnosed, especially if the specimen is limited or from fine-needle aspiration.

## Case presentation

### Clinical history

**Figure 1:**
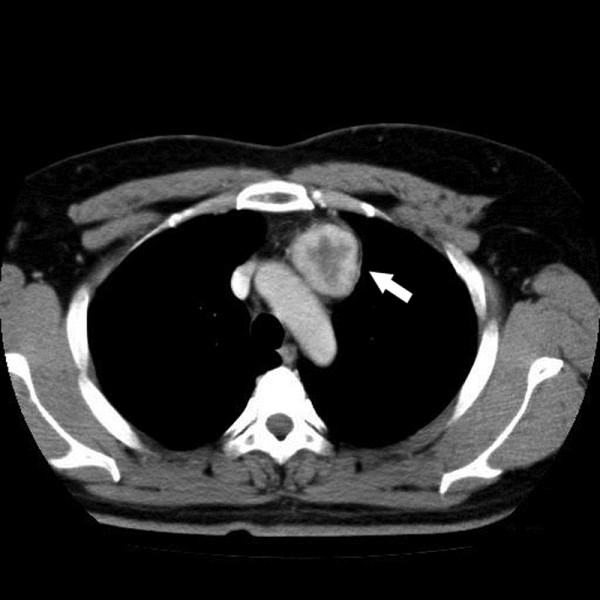
**Oral contrast-enhanced CT scanning of the tumor.** Oral contrast-enhanced CT revealed a well-circumscribed, solitary mass of 4.41 × 3.93 cm in the anterior superior mediastinum.

A 38-year-old male farmer was referred to our hospital for complaining of fatigue and chest distress. No personal or family history was found. Blood examinations were in normal levels. Oral contrast-enhanced computed tomography(CT) scaning revealed a well-circumscribed, solitary mass of 4.41 × 3.93 cm in the anterior superior mediastinum (Figure 1). The tumor was clinically diagnosed as a thymoma, and then a mass excision was performed in our hospital. At surgery, the mass encompassed the innominate vein. The mass was fully removed, and underwent diagnostic examination. According to the morphological and immunohistochemical findings, the tumor was diagnosed as a “high risk” EHE. Then the patient underwent adjuvant chemotherapy. The patient was alive with no tumor recurrence or metastasis within 18 months of follow-up.

## Materials and methods

The resected specimens were fixed with 10% neutral-buffered formalin and embedded in paraffin blocks. Tissue blocks were cut into 4-μm slides, deparaffinized in xylene, rehydrated with graded alcohols, and immunostained with the following antibodies: cytokeratin (CK), CD68,Vimentin, CD31, CD34, CEA, Desmin, thyroid transcription factor 1 (TTF-1), CDX-2, CD5, Calretinin, Actin(SM), CD117, S-100 and Ki67. Sections were stained with a streptavidin-peroxidase system (KIT-9720, Ultrasensitive TM S-P, MaiXin, China). The chromogen used was diaminobenzidine tetrahydrochloride substrate (DAB kit, MaiXin, China), slightly counterstained with hematoxylin, dehydrated and mounted. For the negative controls, the primary antibody was replaced with PBS.

## Results

### Gross features

Grossly, the mass was approximately 4.2 × 4.1 × 3.9 cm, and was relatively well-circumscribed. The cross-section of the tumor was firm and grey-white or grey-red in color.

### Microscopic features

**Figure 2:**
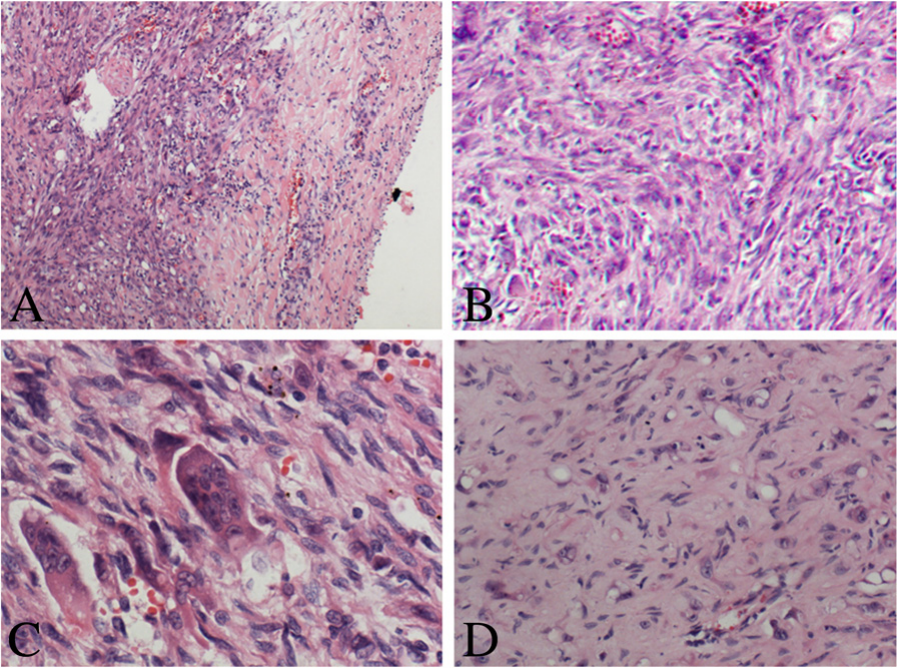
**Morphological change of the tumor. A**, The tumor was relatively well-circumscribed; the periphery may be the remaining blood vessel. **B**, Numerous spindle cells were arranged into sheets or swirling patterns. **C**, The spindle cells had marked cellular atypia, scattered multinucleated osteoclast-like giant cells were present amidst the spindle cells. **D**, The classic histological structure, the cords or nests epithelioid cells with intracytoplasmic vacuoles were focally present in extensive myxohyaline stroma.

Histologically, the tumor was relatively well-defined. The tumor was predominantly composed of abundant plump spindle cells. The cells were diffusely arranged into solid sheets or whirling patterns with little stroma. The cells had moderate to marked cellular atypia, with pale chromatin and conspicuous nucleoli. The mitotic rate of spindle cells was approximately 1 mitosis/10 high power fields. In addition, scattered multinucleated osteoclast-like giant cells were present within the background of the diffuse spindle cells. In focal area of the tumor, the classic histologic structure, the cords or nests epithelioid cells with intracytoplasmic vacuoles in extensive myxohyaline stroma could be seen (Figure 2).

### Immunohistochemistry

**Figure 3:**
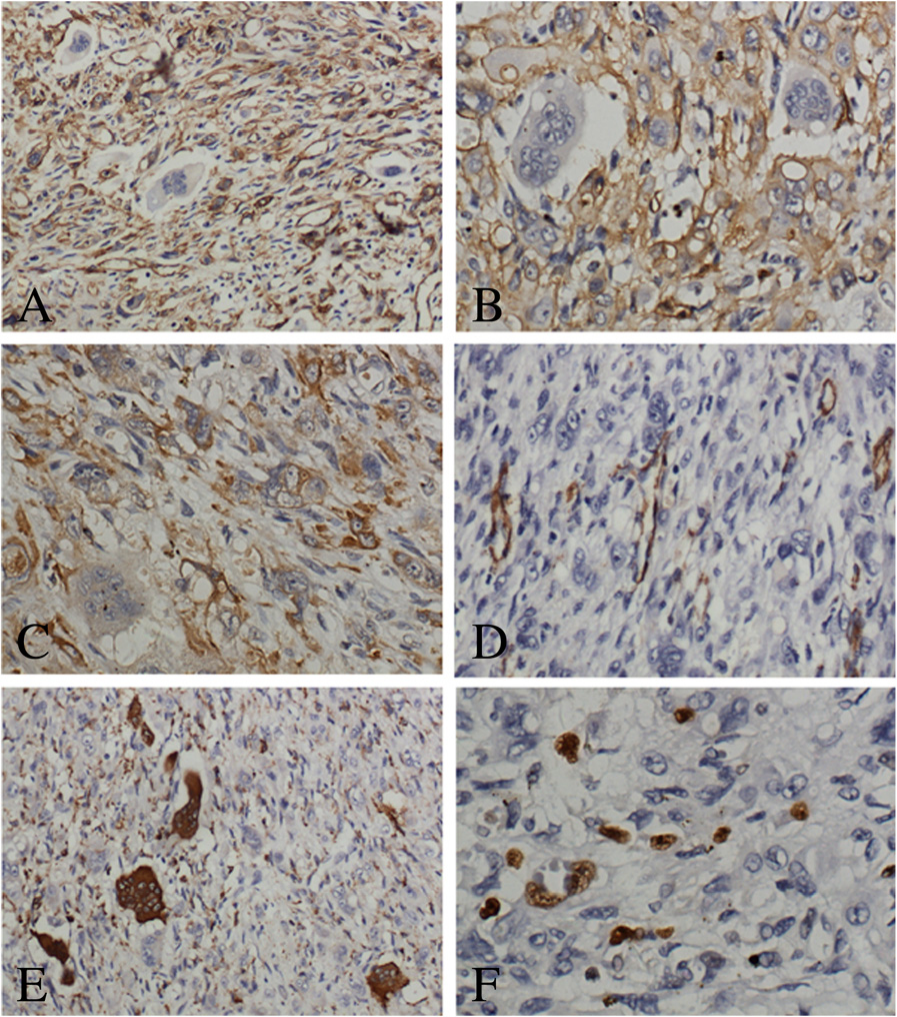
**Immunohistochemical staining of the tumor. A**, The spindle cells exclusive of osteoclast-like giant cells were entirely positive for CD34. **B**, The spindle cells were also positive for CD31. **C**, Diffuse and strong expression of CK could be seen in the spindle cells. **D**, The tumor cells were negative for Actin(SM) in contrast to the positive expression of Actin(SM) in normal blood vessel. **E**, The CD68 staining highlighted the presence of osteoclast-like giant cells. **F**, Ki67 proliferative index was approximately 10%.

Immunohistochemical staining showed that the spindle cells and epithelioid cells were diffusely positive for CK, CD31, CD34 and Vimentin, negative for CD68, Desmin, TTF-1, CDX-2, CD5, Calretinin, Actin (SM), CD117 and S-100. In contrast, osteoclast-like giant cells were positive for CD68, negative for CD31, CD34 and CK. Ki67 index was approximately 10% (Figure 3). According to the morphological and immunohistochemical findings, the tumor was diagnosed as a “high risk” EHE.

## Discussion

Hemangioendothelioma is used to describe a group of vascular neoplasms that may be considered benign or malignant according to their activities. Six histopathological variants have been described: papillary intralymphatic angioendothelioma, retiform hemangioendothelioma, kaposiform hemangioendothelioma, EHE, pseudomyogenic hemangioendothelioma, and composite hemangioendothelioma. EHE is a relatively uncommon vascular endothelial tumor which is considered an intermediate vascular neoplasm between a benign hemangioma and a highly aggressive angiosarcoma. EHE can occur in soft tissues and various organs [5]. Mediastinal location is very exceptional, and only few cases were reported [6-11]. As the most common primary neoplasms in the mediastinum were thymic and neruogenic tumors, the correct diagnosis of EHE is a great challenge.

Histologically, EHE is characterized by cords and nests epithelioid cells with intracytoplasmic vacuoles in a myxohyaline stroma. The formation of intracytoplasmic vacuole represents the primitive vascular differentiation of endothelial cells. Usually, the tumor cells are quite bland, showing light atypia. Infrequently, the tumor cells may be focally spindling, and very rarely, the scattered multinucleated osteoclast-like giant cells may be present within the tumor cells [11-15].

Our case showed extensive spindle-cell changes with scattered osteoclast-like giant cells. Histologically, the tumor was predominately composed of sheets of atypical spindle cells and scattered osteoclast-like giant cells. So, we firstly thought it might be a tumor complicated with multinucleated giant cells such as malignant fibrous histiocytoma. The presence of classic epithelioid cells with intracytoplasmic vacuoles in myxohyaline stroma and the positive expression of CD31 and CD34 can usually favor the correct diagnosis and rule out malignant fibrous histiocytoma [16]. Usually, it is difficult for pathologists to consider the possibility of vascular tumors. If the specimen is limited,from fine-needle aspiration, or histologically lacks the classic patterns, the correct diagnosis may be a great challenge.

In addition, the differential diagnosis also includes some other tumors, which can possess osteoclast-like giant cells, such as giant cell tumor from bone or soft tissue, chondroblastoma, chondrosarcoma, osteosarcoma and dedifferentiated liposarcoma. Based on the histological structure and immunohistochemical staining, the correct diagnosis can be made. Moreover, some sarcomatoid carcinomas can present as extensive spindle cells with osteoclast-like giant cells. Thus, sarcomatoid carcinoma is also an important differential diagnosis. Particularly, in addition to the vascular markers such as UEA-1, factor VIII-related antigen, CD31 or CD34, EHE is also immunopositive for the epithelial marker CK [2], which may be a potential diagnostic pitfall. It is essential to use a panel of antibodies to make the correct diagnosis.

To date, the reported case with osteoclast-like giant cells is exceptional rare [11,13-15]. The significance of osteoclast-like giant cells is still unclear. The osteoclast-like giant cells were only immunopositive for CD68, but negative for CD31 and CD34, indicating these cells may bejust reactive cells. And, further follow-up should be made to investigate its significance.

According to Mentzel et al. [12] and Weiss et al., [17], the majority of EHEs have a relatively better clinical course than highly aggressive angiosarcoma. However, if the tumor shows marked cellular atypia, mitotic activity (>1 mitosis per 10 HPF), necrosis and extensive spindling, it may have a more aggressive course [12]. Deyrup et al. also reported that EHE tumor over 3.0 cm had poor prognosis [5]. But, it is still unclear whether the presence of osteoclast-like giant cells is associated with prognosis. In this case, because of the extensive presence of atypical spindle cells and tumor size, we diagnosed it as a “high risk” EHE.

Treatment of EHE varies and depends on the site and extent of tumor involvement, site(s) of metastasis, and specific individual factors. Surgical resection, radiotherapy, and chemotherapy all have been used to treat these masses, although studies on survival have yet to be conducted to delineate various treatment regimens. Surgery is the preferred treatment as long as the entire tumor could be removed, since there’s little chance of growing back. If it’s impossible to remove the whole tumor surgically, or if there are multiple tumors in several locations, several medications will be commended to slow the growth of the tumor by interfering with abnormal cell growth, ie, anti-angiogenic agent, vincristine, interferon, Rapamycin, radiation and other treatments. The prognosis of EHE is not sound, almost a third of EHEs develop metastases in regional lymph nodes (at least 50% + of all metastatic cases) or in the lungs, liver or bones. Patients who develop metastases have a 50% five-year survival rate. In the current case, the mass has been fully removed by surgical resection, and the patient took adjuvant chemotherapy. Within 18-month follow-up, no recurrence was found.

## Conclusion

Because of the exceptional rarity, the significance of EHE with osteoclast-like giant cells is still unclear. Our reported case was predominantly composed of abundant spindle cells and scattered osteoclast-like giant cells. The unusual histological appearance may pose a great diagnostic challenge, especially if the specimen is limited or from fine-needle aspiration.

## Consent

Written informed consent was obtained from the patient for publication of this case report and accompanying images. A copy of the written consent is available for review by the Editor-in Chief of this Journal.

## Competing interests

The authors declare that they have no competing interests.

## Authors’ contributions

XML, LXY and XHT participated in the histopathological evaluation, performed the literature review, acquired photomicrographs and drafted the manuscript. YJH carried out the immunohistochemical stains evaluation. WL, FCH and LY conceived and designed the study. WEH gave the final histopathological diagnosis and revised the manuscript. All the authors read and approved the final manuscript.
